# Health system barriers influencing perinatal survival in mountain villages of Nepal: implications for future policies and practices

**DOI:** 10.1186/s41043-018-0148-y

**Published:** 2018-07-05

**Authors:** Mohan Paudel, Sara Javanparast, Lareen Newman, Gouranga Dasvarma

**Affiliations:** 1 Initiative for Research, Education and Community Health-Nepal, Kathmandu, Nepal; 2 Southgate Institute of Health, Society & Equity, Flinders University, Adelaide, Australia; 3 Education Arts and Social Sciences Divisional Office, University of South Australia, Adelaide, Australia; 4 College of Humanities, Arts and Social Sciences, Flinders University, Adelaide, Australia

**Keywords:** Perinatal death, Stillbirth, Health governance, Quality of care, Primary health care, Maternity care, Disadvantage, Nepal

## Abstract

**Background:**

This paper aims to examine the health care contexts shaping perinatal survival in remote mountain villages of Nepal. Health care is provided through health services to a primary health care level—comprising district hospital, village health facilities and community-based health services. The paper discusses the implications for future policies and practice to improve health access and outcomes related to perinatal health. The study was conducted in two remote mountain villages in one of the most remote and disadvantaged mountain districts of Nepal. The district is reported to rank as the country’s lowest on the Human Development Index and to have the worst child survival rates. The two villages provided a diversity of socio-cultural and health service contexts within a highly disadvantaged region.

**Methods:**

The study findings are based on a qualitative study of 42 interviews with women and their families who had experienced perinatal deaths. These interviews were supplemented with 20 interviews with health service providers, female health volunteers, local stakeholders, traditional healers and other support staff. The data were analysed by employing an inductive thematic analysis technique.

**Results:**

Three key themes emerged from the study related to health care delivery contexts: (1) Primary health care approach: low focus on engagement and empowerment; (2) Quality of care: poor acceptance, feeling unsafe and uncomfortable in health facilities; and (3) Health governance: failures in delivering health services during pregnancy and childbirth.

**Conclusions:**

The continuing high perinatal mortality rates in the mountains of Nepal are not being addressed due to declining standards in the primary health care approach, health providers’ professional misbehaviour, local health governance failures, and the lack of cultural acceptance of formalised care by the local communities. In order to further accelerate perinatal survival in the region, policy makers and programme implementers need to immediately address these contextual factors at local health service delivery points.

**Electronic supplementary material:**

The online version of this article (10.1186/s41043-018-0148-y) contains supplementary material, which is available to authorized users.

## Background

The concept of the ‘perinatal period’ emerged in the late 1940s after clinicians and researchers became aware of the high level of mortality before and after delivery [1]. According to the World Health Organisation, perinatal mortality comprises stillbirths, i.e. the death of a foetus commencing at 22 completed weeks (154 days) of gestation, and the death of a newborn child within seven completed days after birth [2]. Extended perinatal death comprises stillbirths and neonatal deaths until the first 28 days after birth [3, 4]. The burden of perinatal deaths has inequitably affected developing countries. Almost all (98%) of the total number of perinatal deaths worldwide currently occur in developing countries [5, 6]. Sub Saharan African and South Asian nations have the world’s highest perinatal mortality rates [7].

Health care during the perinatal period, including pregnancy, childbirth and postnatal care, is crucial not only to prevent neonatal deaths but also to effectively contribute to reducing stillbirth as well as maternal death and morbidities. A focus on the intra-partum period—the time between the onset of labour and the birth of a baby—is considered to yield triple returns by preventing stillbirth, early neonatal death, and maternal death and morbidities [8–10]. Perinatal survival including both stillbirth prevention and neonatal survival is the global public health agenda of the Every Newborn Action Plan (ENAP) of the World Health Organisation (WHO) [11]. For the first time, this WHO action plan has merged its agenda for the prevention of stillbirth with neonatal survival, with an aim to end all preventable stillbirths and neonatal deaths by 2030. Ending all preventable neonatal deaths by 2030 is also a health target of the third Sustainable Development Goal [12].

The set of United Nations Millennium Development Goals (MDGs) has recently concluded (in 2015). However, when the stipulated period of the MDGs expired, many countries were far from achieving their set MDG targets, particularly those on improving child survival and reducing maternal mortality (MDG 4 and MDG 5 respectively) [7]. Moreover, in-country sub-regional inequities in health service utilisation and neonatal mortality rates have become further pronounced. Nepal has one of the highest income inequalities in the world [5, 13, 14], and according to 2012 data [5], it was estimated that if the poorest 20% of Nepal’s population had similar risks of neonatal mortality as the richest 20%, then there would be a 46% reduction in the country’s overall neonatal mortality rate. The mortality rates in Nepal’s mountainous region are extremely close to those of the world’s highest rates found in one of the Sub Saharan African countries, Angola, which records a neonatal mortality rate of 49 (per 1000 livebirths) [7]. At the start of the present study in 2014, Nepal’s mountainous region reported a neonatal mortality rate of 46 (per 1000 livebirths) and perinatal mortality rate of over 40 (per 1000 births) [15, 16].

Improving the health and survival of mothers and children is one of the priority areas of primary health care (PHC) in many developing countries [17]. The WHO’s concept of district health systems is the basis for delivering PHC in developing countries, including Nepal [18]. At PHC level, health care in Nepal comprises community-based health services, village-based health facilities and a district hospital [19]. Guided by the comprehensive PHC approach [17], a range of promotive, preventive and curative services are provided for different health problems and diseases. The comprehensive PHC approach considers as key underpinning principles both the local culture and context; community mobilisation, engagement and empowerment; and inter-sectoral coordination. Realising that improvement in health access and outcomes of mothers and their children requires a holistic socio-medical approach, principles and strategies underlying comprehensive PHC are seen as the most effective strategies to tackle ongoing perinatal mortality in both developed as well as developing countries. It is suggested that care across all delivery platforms—outreach, family/community, health facility—is essential to reach out to every woman and child, as a key way to reduce perinatal mortality [9]. Besides focusing on a home-to-health facility continuum, it is recommended that providing maternal and newborn health (MNH) services across a pre-pregnancy to postnatal continuum can avoid any missed opportunities of saving a mother and baby’s life [20]. This evidence about linking community and health facilities, and providing care even before a baby is conceived and born, strongly emphasises the crucial role of a comprehensive PHC approach.

Simple PHC measures are proven to prevent or minimise the occurrence of perinatal deaths. A range of evidence, from developed countries such as the UK and Sweden [21–23], and developing countries such as Indonesia, Honduras, Sri Lanka, Vietnam and Nicaragua [23, 24], shows that successful reductions in perinatal mortality were achieved by reducing neonatal deaths. These studies confirm that a large proportion of neonatal deaths can be averted with a relatively low level of resources by effective community and outreach interventions, such as the use of community midwives and hygiene education, before embarking on more expensive and technical interventions such as the introduction of neonatal intensive care units. Particularly, in high mortality settings, a number of community level interventions have been found to effectively contribute to reducing perinatal mortality [8, 9, 25]. The main purpose of these community/outreach interventions is to target women, their families and communities, and to engage them in health promotive and sickness/disease preventive dialogues. The ultimate purpose is to empower them to have control over their health and survival, with access provided to skilled professional care when needed.

*The Lancet* series on neonatal and stillbirth survival between 2005 and 2016 [26–29], and the 2015 *BMC Pregnancy and Child Birth series* on ‘Every Mother Every Newborn’ [30], discussed in length the available interventions on the pre-pregnancy to postnatal continuum, and the success of simple PHC measures in reducing perinatal death. Two key messages from these series are as follows: (i) further improvement in perinatal survival in developing countries requires a shift in focus from only a high-tech medical approach to include interventions at home, community and health facilities; and (ii) priority attention is needed to address health system constraints which hinder scale up and effective implementation of the available interventions. Therefore, it is not merely the lack of interventions and strategies that has kept perinatal mortality persistently high in African and South Asian countries. Rather, it is also a lack of effective implementation of available interventions, which are estimated to reduce over two thirds of current neonatal mortality [8], and similar reductions in stillbirth [8–10].

Studies previously conducted in Nepal have a limited focus on understanding how the health service delivery contexts shape perinatal survival at the PHC level. A range of studies have described medical causes and neonatal mortality distribution across socio-demographic attributes, predominantly from Nepal’s plains (*Terai*) and semi-urban hilly regions, which have comparatively better physical and social access to health care information and services compared with the mountainous region [31–42]. Some of these studies only used medically focussed verbal autopsies which describe infection and intra-partum-related conditions, such as asphyxia and obstetric complications as key reasons for perinatal deaths [38–42]. Some are cross-sectional quantitative surveys limited to describing the pattern of service utilisation for pregnancy, birth and postnatal care [31–37], and none of these studies cover the mountain region. Other studies, based on national demographic and health survey (NDHS) data, describe the pattern of neonatal mortality and its distribution across the nation [15, 43]. The NDHS report shows the level of neonatal mortality as being high among the rural, poor, low educated, disadvantaged and indigenous caste/ethnicity, and in the mountainous region [16]. However, both the literature and available government reports lack an in-depth examination of health service contexts in relation to pregnancy, childbirth and perinatal health. Therefore, this study aims to examine the health service delivery context at a PHC level in the mountainous region, which continues to report the highest perinatal death rates in Nepal.

## Methods

### Study design and setting

This study is guided by social constructionism and critical theoretical approaches to examine how the context of health care delivery contributes to poor perinatal survival. Social constructionism views knowledge as culturally situated; local, specific and co-constructed realities with consensus among those engaged in construction; and aiming to reveal or understand the phenomenon [44, 45]. Studies with a critical theoretical perspective have a focus on social justice [46, 47] and hence are relevant to the aims of this study which intends to provide new information to address the poorer levels of health experienced by the country’s most disadvantaged populations. Both social constructionism and critical theoretical perspectives allow dialectical methods to collect data; therefore, qualitative interviews were considered appropriate in this study. A qualitative methodological approach allows for the voices of unheard, silenced and marginalised groups [48, 49] to be heard and for an understanding to be developed of the study participants’ experiences and meanings in natural settings in a sensitive manner [49], considering that the mothers who participated in the study had all experienced perinatal deaths.

**Fig. 1:**
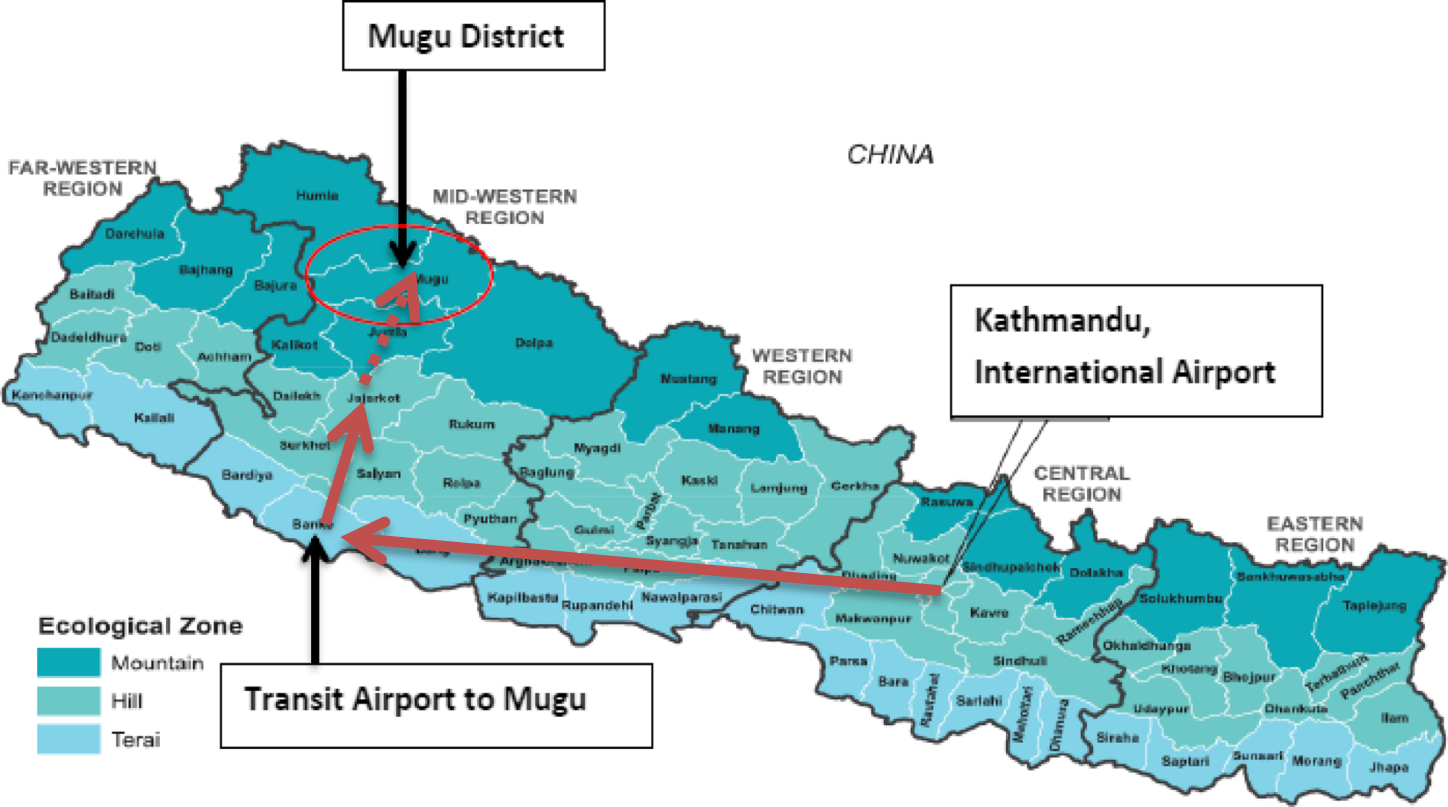
The study district in Nepal’s map, situated in the west of Nepal, and bordering Tibet in the north

**Fig. 2:**
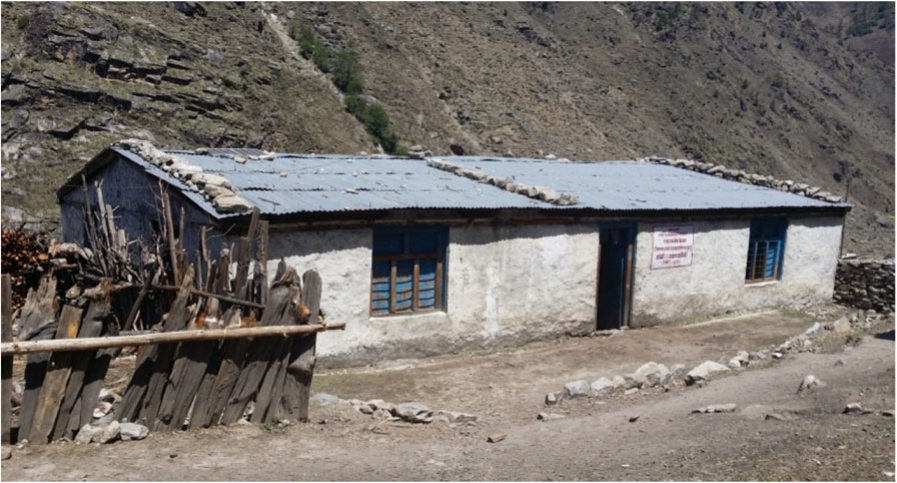
Health facility (birthing centre) in the second village (photo credit: first author)

Two study villages were selected from Mugu, a district in Nepal’s remote mountainous region (Fig. 1), because in Nepal this district ranks the lowest on the Human Development Index in the nation (at 0.304) [50]. The study villages were purposively chosen based on accessibility for the researcher, religio-cultural composition, population size and availability of childbirth services. The district covers an area of approximately 3500 km, with a population of 60,000 and 24 villages. The mountainous region is reported to have the highest perinatal/child mortality rate in Nepal [16]. Health care in the villages is provided through a network comprising one district hospital, a local health facility in every village and seven birthing units. Health care is extended at family/community level through a network of PHC Outreach Clinics (ORC), female community health volunteers (FCHVs) and Mothers’ Groups. The first study village had approximately 4000 inhabitants primarily following Hindu religious beliefs; they had access to a birthing centre, a local health facility and a nearby district hospital (at a walking distance of less than 1 h). The second village comprised approximately 3000 inhabitants following both Hindu and Buddhist religious beliefs; it was at a walking distance of 7 to 8 h from the district hospital for 8 months of the year and over extremely steep terrain (inaccessible October to January, even by plane), but had a local health facility staffed by auxiliary nurses and assistant health workers with a birthing unit (two basic rooms, very limited equipment not always in working order, and no indoor water supply, heating or lighting) (Fig. 2). Neither village had a reliable electricity supply. The villages were chosen as they had a relatively larger population than other villages of the study district, and compared to some other very high altitude villages, they had a generally low seasonal migration of people [51, 52], which enabled access to reach the required number of women who had experienced a perinatal death in the previous 4 years.

### Data collection and participant recruitment

Data collection included qualitative interviews with women and their family members, health service providers and stakeholders. The interviews were conducted using in-depth interview guides prepared specifically for each group (Additional file 1). The interviews solicited the women’s and their family members’ views about perinatal sickness and deaths, and their experience of interactions with health service providers and female health volunteers in local health service delivery settings. The interviews with health service providers, local stakeholders, support staff and traditional healers were aimed to obtain information about care availability, utilisation and constraints in accessing health care by mothers and their babies.

The women’s interviews (*N* = 42; Table 1) were supplemented by interviews of the 11 health service providers (10 nurses/auxiliary nurses, 1 Auxiliary Health Worker), two FCHVs, two support staff, one traditional healer and four local stakeholders, and field notes (Additional file 2).

**Table 1:** Socio-demographic characteristics of the participants (women and family members, *N* = 42)

Attributes		Number of participants
Age (current, completed years)	15–20	17
	21–35	24
	36 and above	1
Age at marriage (completed years)	< 15	10
	15 to 20	30
	> 20	2
Education (Women)	Illiterate	29
	Just literate (no formal schooling)	3
	Primary level	2
	Secondary or above level	8
Education (husbands)	Illiterate	13
	Just literate (no formal schooling)	5
	Primary level	5
	Secondary or above level	19
Religion	Hinduism	32
	Buddhism	12
Caste	Lower caste	12
	Upper caste	32
Number of Pregnancy	Up to 2	19
	2 to 5	17
	> 5	6
Main source of income	Day-to-day labour work	13
	Agriculture	27
	Service (government/non-government employment)	2

Local health service providers, the traditional healer and local stakeholders were recruited by the first author. The service providers, traditional healer and local stakeholders were either involved in managing or providing modern/traditional maternal and child health services in the villages. The sampling strategy to recruit women participants comprised a blended approach of priori sampling [53] with specific criteria, and a theoretical sampling approach [54]. The recruitment criteria were as follows: any woman from the study villages who had experienced a perinatal death in the last 4 years (but not within less than 2 weeks out of respect for cultural/ritual reasons in the Nepalese context; and not earlier than 4 years previously in order to minimise bias due to mother’s recall) and who expressed her willingness to participate in the study. A woman who had experienced perinatal death was defined as having either had a stillbirth after 22 completed weeks of gestation/six completed months (whichever the mother could recalled), and/or a neonatal death during the first month after birth. The number of women participants was decided theoretically, when the first author noted no significant new insight emerging after the first 37 interviews; the additional interviews provided a sense of saturation of the study phenomenon, i.e. the health service delivery context of perinatal death in the study villages.

To assist in recruiting women from the villages, local female health volunteers, a social mobiliser and a health worker were utilised as gate keepers. According to Devers and Frankel [55], utilising such gate keepers effectively facilitates the recruitment of participants and creates trust with them. A few participants were also recruited through participants’ contacts (snowball sampling [56]). Fieldwork for the data collection was carried out between February and June 2015. Data from the participants’ interviews were supplemented by field notes taken during the interview, post-interview informal chats and observations made by the first author while visiting local health facilities. Interviews were conducted by the first author, who is a Nepali national with over 7 years of experience in working in the maternal and child health sector in Nepal and fluent in the local Nepali language. The interviews were digitally recorded with participants’ consent. None of the family members were prevented from adding their experiences during the interviews with the women or afterwards. Interviewing at home was culturally more acceptable than interviewing at other places. Rather than conducting a separate interview away from home, the home setting together with family members comforted women and their families and was culturally respectful. Interviewing a woman who has experienced perinatal death is potentially a sensitive topic (from a Western perspective). However, not a single woman reported distress or seemed distressed, because perinatal deaths are considered such a natural occurrence to everyone; indeed, women rationalised such deaths as due to their *Karma* (past deeds), *Bhagya* (fate) or *Lekhanta* (destiny) [57]. Indeed, participants felt rather proud to have someone (the first author) coming to their doorstep to listen to how they went through these experiences. Due to such enthusiasm to share their stories, the first author also felt it important to extend an interview to a few women even though they had had post-neonatal infant/under-five deaths, but these are not included in the data in this paper.

### Data analysis

The interview recordings were simultaneously translated and transcribed into English by the first author. To ensure translation rigour, six random interview records were sent for language check to bilingual experts, who were doctoral students and research fellows at Flinders University. The data were analysed using the thematic analysis technique as suggested by Braun and Clarke [58]. Thematic analysis is a qualitative research analytical technique to identify, analyse and report various themes in the data. In this study, themes are the key concepts about data, which reveal the health care contexts influencing poor perinatal survival in the study villages. The Braun and Clarke process was followed in terms of conducting a thematic analysis in six phases: familiarisation with the data including transcribing, generating initial codes, searching for themes, reviewing the themes, defining and naming the themes and writing a research report. NVivo software version 10:00 was used to organise data and facilitate the coding process in this study.

### Ethical considerations

The study received ethical approval from the Social and Behavioural Research Ethics Committees of Flinders University, Australia (project no 6702), and the Nepal Health Research Council (Reg no 271/2014). The participants were recruited in this study with their voluntary and informed written consent.

## Results

Three key themes emerged from this study which are related to the local health service delivery approach, the quality of care as experienced by women and local health governance.

### Primary health care approach: low focus on engagement and empowerment

The approach to health care delivery in the study areas lags far behind the standards of comprehensive PHC, which should be integrated (including preventive, health promoting and curative aspects), inter-sectoral and people-centred [17]. The interviews in this study revealed that health care is limited within health facility premises and that care is limited to the provision of basic medicines in the form of pills.

#### Becoming a ‘doctor’ and prescribing pills

Health care, from the viewpoint of the PHC providers, is perceived only as medical care. A doctor in these communities is valued as someone who speaks little with clients, stays in a health facility, checks a patient with a stethoscope (*Ala*) and prescribes pills. This is far from the principle of comprehensive PHC which emphasises community involvement, integration, empowerment and health promotion as key principles [17]. Moreover, all the PHC workers interviewed, including a support staff member and FCHVs, are happy to be called ‘doctors’ by the patients attending the health facility, even though none of them is a qualified medical doctor. This has helped propagate a false image of all the auxiliary staff members as “doctors” among the patients. One of the community health workers summarised the situation as follows:

Everyone (other health workers) including a female health volunteer is [considered /seen as] a doctor here. This is the image that has built-up for a long time in our society. They are not interested in preventive and promotional activities. They feel themselves to be like a medical doctor when they can prescribe pills, and put *Ala* (stethoscope) on a patient’s chest. (Community Health Worker, HSP15).

It was found during the interviews that women in the villages call any male health worker, including a member of support staff, such as a peon (orderly), a doctor (*Doctar* or *Doctorsab*), and a female health worker including an FCHV, a female doctor (*Doctarni* or *Doctornisab*). The local terms, particularly *Doctorsab* or *Doctornisab*, are terms denoting a higher social status than that of the women and families in the communities. The perpetuation of this image has reinforced among the auxiliary health workers a fake self-perception as being doctors who are more highly qualified than they actually are, and they have redefined their roles on their own. The quote below describes a situation where villagers simply aim to receive pills *(Tata)* as health care, and a peon (an orderly) accepts the false image of a doctor and values prescribing pills from local health facilities:

Villagers come early in the morning asking for pills *(Tata)*. We don’t need to open the health facility during the daytime; no one comes during the daytime. They themselves say, ‘why do you suffer coming here during the daytime? You can give us pills early in the morning and then you can go back, that is okay’. (Support Staff, HSP6).

During a post interview chat, it became clear that the peon feels proud of his ability to prescribe iron pills, anti-worm pills and even tetanus toxoid (TT) injections to pregnant women. The peon is not a qualified health provider. S/he is a staff member who works as a security guard of a health facility.

The perception of health care as solely the provision of basic medicines has been transferred to community members. A health facility has turned into a drug (medical) store, understood by the villagers as a place to receive pills. However, a few women and families feel unsatisfied when they are not provided enough information about the causes of the mother’s and baby’s sickness. A young man described his sad experience of seeking care from the district hospital after his wife’s first pregnancy ended in a stillbirth and the unhappiness about the treatment received for his sick baby during his wife’s second pregnancy:

Sometimes, they [local health workers] say they don’t have pills *(Tata)*. If they don’t have pills, why can't they educate us? Why don’t they tell us about what has happened to our babies? It is so sad. We just have to return without any care; they don’t talk to us about the sickness. If we go to the hospital, they merely touch you with their hand and put *Ala* [stethoscope], they don’t explain the problem, and hardly give us even one tablet. We have to go to drug (medicine) sellers anyway [to buy more pills]. So, now I feel that if I have money, it is better to go directly to these local medical stores. (26-year-old father)

The doctors or auxiliary health workers do not engage with the women and their families to give them health education and counselling. As described above, health promotion and prevention at the primary healthcare level are considered as major activities, both internationally [17], and also nationally as stated in Nepal’s national policy documents [59, 60]. However, on the ground in these villages, it is considered that dispensing pills is all that healthcare consists of, including the care related to the FCHVs. Female community health volunteers are considered as pillars of Nepal’s community health system [61], yet the critical role of the FCHVs as community mobilisers to empower women to demand skilled care, as intended in national policy documents [62], is limited only to distributing pills. One of the FCHVs stated:

We give them pills [anti-worm pills, vitamin B complex and Iron]. Yet, they continue losing babies. We don’t have anything more [medicines] to prevent these losses. We only get a few pills. (FCHV, HSP11).

#### Home and community visits as mere slogans

Provision of health services through home/community outreach visits occurs rarely in the study villages, although in the national policy document this is described as one of the main roles of health workers [59]. Health workers simply wait for women to come to the health facilities for their pregnancy, childbirth and postnatal check-ups:

We call women to the health facility here. We don’t go to attend homebirths. Only women from nearby [from only one settlement out of the five settlements in this village] come to contact us. We don’t know what is happening in the other settlements. This month, there has not been a single woman coming for birth in this health facility. (Auxiliary Nurse Midwife, HSP8).

Despite the national policies discouraging homebirths [60, 63, 64], almost two thirds of women still give birth at home in these regions [65]. A safe delivery kit (SDK) was previously provided to ensure safe and hygienic homebirth and to prevent infection, one of the most common causes of neonatal death. However, the strategy to discourage homebirths includes the discontinuation of the supply of safe delivery kits to the villages 7 years ago, as explained by an FCHV:

We got the Kits [Safe Delivery Kits] just once from a non-governmental organisation. It is about seven years since they stopped supplying them. Women shout at us, ‘You gave us Kits before, but not now, why?’ They don’t supply these Kits now. (FCHV, HSP11).

A member of the support staff from the health facility explained that they report the outreach service as being functional even though it is largely neglected:

I have never seen any health worker coming to our community to conduct the Outreach Clinic. The health institution has declared my house as a centre for the Outreach Clinic. The medicine box [first aid and basic drugs for outreach services] has been stored there for more than one and a half years. They (health workers) say they conduct outreach…, They just lie about it, merely reporting on paper. It is dishonest. (Support Staff, HSP6).

Another local auxiliary nurse said that she had only run an ORC once in the past 2 years of her work in the health facility. As per the local health facility target, there are three to four such clinic sites in each village; each should operate at least once every month. By comparison, in reality, the value of the outreach service has been reduced to a ‘box of pills’ containing some pain medication, antacids, packets of oral rehydration solution and medications for diarrhoea control and skin infections. Routine outreach services are supposed to counsel and educate women and families about family planning, pregnancy and postnatal care, conduct immunisation, and treat common diseases such as pneumonia, diarrhoea and wounds [65].

Women in the villages are therefore at a double disadvantage: they have received neither any care from the community outreach nor any quality care from health facilities. ORCs are not functioning, yet in the monthly service reports, these are indicated as being functional. Care from health facilities before and after childbirth is limited to recording the number of visits (contacts) such as antenatal and postnatal visits, or giving out pills as described above. Provision of quality pregnancy and childbirth services and empowerment of the village women and families to demand and control health care and survival of their own and their children are lagging far behind compared to what is aimed at in Nepal’s national policies [59, 60, 63] and through community-based interventions [9].

### Quality of care: poor acceptance, feeling unsafe and uncomfortable in health facilities

Quality of care has multiple aspects such as safety, effectiveness, timeliness, efficiency, equitability and the ability to be people-centred [66, 67]. The World Health Organization’s health system approach suggests that quality in maternal and newborn care could be examined from two dimensions: provision of care and experience of (receiving) care [67]. This section examines quality of care in terms of women’s experiences related to safety, comfort and respect in the health facilities, and their acceptance of recommended newborn care.

#### Care of a newborn baby: poor acceptance of the recommended care

This section highlights a significant misalignment between the policy recommendations and what participants in the study villages considered as appropriate for the care of their newborn babies.

##### Baby is not clean and active unless bathed

Postponing bathing for at least the first 24 h after birth and keeping the newborn baby warm with soft and clean clothes are the national policy recommendations to prevent hypothermia [59]. However, bathing a baby immediately after birth is a common practice in the study villages because the comfortable and cosy environment in the mother’s womb is considered to have made a baby lethargic (*Astadiyeka*). Therefore, an immediate bath with cold water (usually outside the house, but only in spring summer and autumn when the temperature would be approx. above 10 degrees Celsius) is perceived to make a baby active and initiate crying. Bathing is also considered essential to clean a newborn’s skin and make a baby look good. A pregnant woman with her 10th child said:

When we bathe them with cold water, they will be active and you can hear baby’s cry *(‘Umm Gaa’)*. This makes the baby healthy and appears good. So, immediately after birth *(Hudaina Sahita)*, it is good to bathe them with cold water… Baby is covered with the dirty thick white substance (*Leiu*: vernix), sometimes also blood in it; it looks filthy *(Sisiko)*. This can infect the skin *(Chhala Pakne)*. (35-year-old woman)

Drying a newborn with a cloth is considered invasive to their delicate skin, and therefore, a soap and water bath is considered appropriate. Women also do not like the appearance of their baby’s skin and its smell until their babies are bathed.

Villagers also reported bathing apparently stillborn babies to see if the babies would cry and might still be alive (*Paran*)*.* There were stories of stillborn babies evidently coming to life after being given a bath. A woman reported that she bathed her baby even though it was stillborn:

We also bathed my first baby [stillborn] immediately after birth. This can wake up babies and help them breathe if they are still alive anyway. We bathed the baby three times. (28-year-old woman)

Women are also hesitant about drying the baby instead of bathing, which is a practice in health facilities. This was one of the reasons for the women’s low preference to attend health facility births. The lack of basic amenities in the health centres, such as showers and washing facilities, was another factor preventing women going to give birth in health facilities.

##### Newborn babies are too young to wear clothes

Clothing babies immediately after birth is recommended to prevent newborns from getting hypothermia [59]. The newborn care guidelines in Nepal recommend baby clothes such as trousers, woollen caps and socks (*Bhototopi ra Moja*) [68]. Nevertheless, the village women and their families do not consider it worthwhile preparing clothes for newborns until they feel confident about the babies’ survival. Most families just use a piece of old cloth, locally described as *Talo*, to wrap their babies in after bathing. The *Talo* is usually a torn portion of the mother’s old sari (*Dhoti*) or shawl (*Barko*)*,* or of her cloth belt (*Patuka*)*.* A young mother who lost her two newborns explained:

These *(Talos)* are pieces of my sari *(Dhoti)*. I used them to wrap my babies in after birth. When a baby is born, we get these saris, tear them and wrap the baby with it. We don’t buy any separate clothes [like trousers, caps]. Newborns are very young to put clothes on. (25-year-old woman)

Since a newborn baby is seen as too young to have clothes, some women and their families were surprised when they were asked whether they had clothes for their newborn after birth:

That was a very young newborn [died on the 7th day after birth in *Gotha*], why does he need clothes? [Surprised]. We don’t know whether the newborn will survive. We don’t buy clothes until a baby starts crawling, better to buy when they start walking. (18-year-old woman)

The research also revealed that baby boys in some families are more likely to have clothes earlier as gifts during *Chhaith*, a sixth day ritual after birth*.* Yet, they do not like to use these clothes until the baby grows older. For a baby to wear new clothes early after birth is believed not to bring good fortune, something which the FCHVs also believed.

##### Delayed breastfeeding

It is recommended that babies should be breastfed immediately after birth [69]. This helps to establish a bond between the mother and baby, the baby learns to suckle mother’s milk, and it also prevents neonatal hypothermia. However, in the study villages, it is uncommon to breastfeed a newborn before the placenta has been delivered and disposed of (*Salnal Saffa*), and before the baby has been bathed. One woman said she could not breastfeed her baby throughout the day as she had to wait until midnight to deliver her placenta:

I delivered her early in the morning at about 7:00, but I couldn’t deliver my placenta until midnight. I couldn’t breastfeed baby before this. (22-year-old woman)

During the fieldwork, even the FCHVs in the villages postponed breastfeeding until the disposal of the placenta. During informal chats after interview, it was revealed that further delay in breastfeeding occurred because the women were not allowed to wash their clothes or shower in the regular taps/wells. As women after birth are considered polluted, they can take a shower only in selected taps/wells, which are usually located far from other taps/wells and can take 10 min or even longer to reach on foot.

The first breast milk (colostrum) is highly nutritious, contains all the nutrients required for an infant for up to 6 months and protects the baby from common problems such as diarrhoea and pneumonia [69]. Colostrum is highly nutritious and has an anti-infective property and is rich in protective factors [69]. However, avoiding colostrum is a common practice among mothers and their families in these villages. When a young woman lost her newborn due to diarrhoea and vomiting, she perceived that her baby’s sickness was due to indigestion of her first milk (colostrum):

The nurse in the hospital told me to feed the first milk (colostrum). But, I think it was not good for the baby, difficult to digest it. My baby vomited the same milk, and also had diarrhoea *(Dudh Ukhelne and Chherne)*. (20-year-old woman)

#### Feeling uncomfortable and unsafe in health facilities, freedom and ease at homebirths

The participants in this study reported experiences of fear of, and mistreatment from, service providers. They perceive providers’ behaviour as controlling, and making them feel hopeless and frightened during childbirth. Those women, who had delivered their baby at a health facility once, did not like to repeat it because of their unhappy experiences:

I gave birth to all my children at home except this daughter. I did not feel good in the hospital. They [nurses] just throw us in bed. We can’t even move our body;… They shout at us, ‘Do this, bitch (*Randi*)’. I was afraid at the hospital. I was worried that if I died there, I wouldn’t see my children or my neighbours again. At least we could die easily at home… During the last birth, I was lucky; God helped me to deliver on the way [to hospital]. This time, I will try to deliver at home until the last minute. (25-year-old woman)

Mistreatment by health providers was the most commonly raised concern by women that made giving birth at a health facility a low-level preference. This disinclination to attend a health facility for birth is also related to the perceived small amount of monetary incentive offered by the government, which is 1500 Nepalese Rupees (about 15 USD). One study participant, a woman pregnant with her 10th child, who lives at less than an hour’s walking distance from the hospital and less than 20 min’ walk to the local birthing centre, stated:

What to do with Rupees 1,500 [about 15 USD]. This is a tiny amount. This is okay just to buy one small plate *(Bila)* of meat. The nurses are not good *(Bichkiyeka)*. They shout at us. I can give birth by myself here [at home]. It is much better at home. (35-year-old woman)

The sense of feeling unsafe during birth in a health facility is also linked to the women’s experience of not being able to save a previous baby even after giving birth at a health facility:

My previous two childbirths were born in a hospital. Both times, I lost my babies. Therefore, I didn’t like to go there last time. This baby [the third child] was born here [at home], and he is alive. (19-year-old woman)

During post-interview chats, it was found that the feeling of being unsafe at the health institution is also related to the use of augmentation for labour the women’s concerns that augmentation could cause their baby to die.

While the women generally felt that they are ignored by the hospital staff (doctor, nurses and other members of staff), both the women and their families also had doubts about the providers’ competence in treating the mother and her baby should they fall sick:

They don’t care about us in the hospital. We are just ignored. And, you know, there are non- experienced health workers. I think they don’t know much. (21-year-old man)

I didn’t like to go to hospital. I lost my two children there. We stayed over a month for the treatment of our second baby. Health workers said that our baby got pneumonia. It didn’t improve, he died in hospital. Last time [during her third pregnancy], I didn’t like to give birth in hospital. Also, this time I will not go there, I will give birth at home. (A 20-year-old woman)

Another family was seriously concerned when a health provider injected their newborn at several sites in his hand, attempting to attach an intravenous device for administering medication. This family discontinued the child’s treatment after the first 3 days and went home despite the health provider’s advice to stay in the hospital. The mother-in-law of the child’s mother commented:

They injected here and there [punctured many times in baby’s arms] to that little newborn. There was blood from the injection sites in the hand. Baby’s hand was swollen due to the injections. We did not feel good staying there. We brought the baby home. On the following day the baby died. These health workers can’t save babies from dying. (Grand mother-in-law, 68-year-old woman)

### Health governance failures in delivering pregnancy and childbirth services

Nepal has experienced a number of policy updates to ensure nationwide access to 24-h childbirth services and round the clock basic and comprehensive obstetric care [59, 60, 64, 70]. Yet, a range of health system failures were identified in these villages which hinder the delivery of even basic care during pregnancy and childbirth. Several issues, particularly rural workforce support, and the lack of local health system accountability, emerged as key factors hindering access to pregnancy and childbirth services in the study villages.

#### Health workforce support: lack of an enabling work environment for skilled birth attendants

Nurses and auxiliary nurses comprise the major bulk of the SBAs working in rural areas of Nepal. Nepal’s national SBA policy considers provision of care during pregnancy and childbirth as the key duties of the SBAs [63]. An enabling environment for these providers is crucial to ensuring their high performance. Personal and family issues related to staff safety, security and loneliness; support from co-workers and the community; and the availability of accommodation and basic amenities for living in the health facilities were the key issues in workforce practice for the SBAs. In the study villages, most of the 42 women interviewed had never had access to a SBA.

There are many factors contributing to the lack of safety among nurses working in the rural areas. These include the local men’s drinking habits, and a lack of basic living amenities available, such as safe quarters, water supply, communications and electricity in the health facilities:

There are no quarters. I am living in my house. At least, I am a local. But, it is almost impossible to live and work here for the nurses coming from other villages or from outside the district. Because these are *Bhote/Lama* communities, they drink a lot of alcohol. Even if they are good when sober, they get mad after drinking. So, you don’t feel safe here. (Nurse in Lama community, HSP5).

A senior auxiliary nurse from the hospital described a recent incident of attempted rape by a local village teacher on another auxiliary nurse, which resulted in the auxiliary nurse leaving the village:

You know, just a few months back, a new auxiliary nurse came here. She went to the village and rented a room in a villager’s house near the health post. One day, a man broke into her room and attempted to rape her. …she was too scared to stay in the village. So, she took a transfer from the village after two months. (Senior Auxiliary Nurse Midwife, HCM3).

On observation, there was no staff accommodation in the first village birthing centre, and the accommodation provided to the nurses in the second village was a small room with no secure door or window. The toilets were not functioning. Moreover, accommodation had neither running water nor cooking facilities. The nurses had to fetch firewood to cook and get buckets of water from a tap at some distance from the health facility.

In addition to poor living conditions and safety issues, working as a sole health worker in the village health facilities imposes a high workload. Nurses felt burnt out due to the 24-h working shifts and lack of any time for their families:

You have to do night, morning, day and evening duties on your own. Yet, you don’t get any additional incentive for it. Our family members are not happy with it. They frequently complain to us, ‘You are doing 24-hour duty. How much are you earning then? There are school teachers who work just 10am to 4pm and earn the same. You don’t have any spare or time even when someone in our family is sick’ (Auxiliary Nurse Midwife, ANM 7).

In village health facilities, lack of team support for a nurse makes them feel alone and hopeless while managing a mother and child’s complications. One of the nurses shared her experience about resuscitating a newborn baby when she felt nervous and had no co-worker support:

I was alone in the health facility. After birth, the baby was asphyxiated. We didn’t even have a bag and mask in the health facility. I tried mouth to mouth respiration, but couldn’t save that baby. I was nervous; I requested my co-worker (auxiliary health worker), but he didn’t support me. Then, I called a traditional birth attendant. At least, I didn’t feel alone with this traditional birth attendant. Unfortunately, we couldn’t save that baby. I still remember this today. These auxiliary health workers don’t support us in childbirth even if we are alone. They do not consider it to be their job. (Auxiliary Nurse Midwife, HSP4).

Due to these poor living and working conditions at the village health facilities, nurses often want to flee from the village to the district hospital. Women and their families who often seek health care at a late stage of sickness expect a solution for any of their health problems from the local health facilities. They are reluctant to go to higher levels for care even if they are advised by the local nurses. Such a high level of expectations by the village women from nurses on the one hand, and the lack of work place support, safety and basic living conditions on the other, has demotivated nurses working in village health facilities.

#### Pregnancy and childbirth in a poorly accountable health system: negligence, lack of monitoring and corruption

Negligence, lack of a monitoring system and corruption emerged as key factors hindering delivery of basic health care services during pregnancy and childbirth. Women do not see any point in going to the health facilities as these are frequently closed during the daytime. They explained:

There is no point in going to the health facility. I don’t go. No one comes there. This is shut during the day. Every day is a holiday for these health workers. They just come once a month for immunisation. (26-year-old woman)

When I had intolerable labour pain *(Kaitha)*, I went to the birthing centre (*Bhavanghar*). It is just over there, takes only five minutes. However, it was closed. I didn’t see anyone there. (25-year-old woman)

It is almost impossible for women to expect the presence of health workers during the weekend, and at night-time on weekdays. Therefore, going to a health facility for childbirth is a big disappointment for the village women:

I had labour pain at night. There were no health workers in the health post. Where to go at night? Neither the male health worker *(Doctor)* nor the nurse (*Doctorni*) stays there. They just occasionally show up at the health post. (22-year-old mother)

In the first village, it was the male peon—an orderly in the health facility—who occasionally opens the birthing unit. Health workers are absent from the workplace, and it is the male peon who works as a birth attendant:

I have attended many women alone in this birthing centre. You know, I had to attend two women in the front yard [he points out to the front] when they were at the last stage. I also delivered a woman in the toilet and another in the children’s play room. I was not given the key to the labour room. Some women simply returned home. Later, the nurse (*Madam*) and the auxiliary health worker decided to give me the labour room key as well. Now, I can attend to any woman. (Support Staff, HSP9).

It was also noted that the service providers are present at village health facilities only towards the end of each month just for the sake of lodging their monthly progress report to get their pay released from the district:

The [person] in-charge appears at the health post when it is time to send the monthly progress report to the district office. We have one person in-charge (health assistant) of the health post, one village health worker, one auxiliary nurse and two auxiliary health workers. However, they are there only on paper. They are not physically present here. It is only me opening this health post. All other staff are busy in their private medical stores and household work. (Support Staff, HSP6).

One of the local journalists also commented on health service providers’ lack of responsibility towards public health facilities:

They do not stay in the village heath posts. They receive monthly pay from the government. But, they are always seen busy in their [private business] medical stores. (Local Journalist, SH2).

Absence of staff from work was also common at the District Health Office (DHO) level which is responsible for supervision and monitoring of village health facilities. It is many years since the district office has had a medical superintendent, the person in charge of the district. The district hospital initiated caesarean section (CS) in 2013 with a team of volunteer nurses and a gynaecologist from an international non-governmental organisation (INGO). However, it was soon discontinued when the doctor and nurse who were supported by the INGO left the district. Also, during the field work for this study in 2015, the first author met a gynaecologist and nurse from the INGO who had been there for months to support the resumption of CS. However, the hospital was not able to resume CS by the end of the study fieldwork as they could not arrange an anaesthetic assistant. A complicated scenario was also related to false reporting of health facility births and misuse of incentives. In the first study village, during a review of local birthing centre records, more than half the births were falsely reported as having occurred at the birthing centre.

##### Misuse of childbirth incentives

The national health policy outlines that women attending health facility births will receive a childbirth incentive immediately upon their discharge to compensate for their transportation costs [64]. However, it is evident from conversations with various people that women in the villages had not received their incentive payment for more than 2 years after delivering their babies at health facilities:

It is already two years since less than half of the women have had the incentive. It is not clear when the nurse (*Madam*) brings and distributes it to them. It is up to her. (Support Staff, HSP9).

The journalist who was interviewed also mentioned that he was threatened a few times by local health workers when he wrote in local and national newspapers about the misuse of these incentives and the absence of health workers. Reports of health workers misusing childbirth incentives were also frequently heard during the fieldwork. During informal chats, local leaders also talked about misuse of childbirth incentives, the closure of health facilities during the day, staff absenteeism and stories of mother and child deaths. However, they lost sensitivity towards such issues and so preferred to remain quiet.

Another nurse related problems due to the staff’s lack of accountability and corruption in procurement and supply:

Look, even here at the district hospital, we are disappointed. It is a shame. We ran out of threads to tie baby’s umbilical cord, no cord clamps. We made the threads from gauze. I requested to make a purchase from the store section time and again. But they don’t care about it. This system is corrupted because they are just making bills without making any purchase. (Senior Auxiliary Nurse Midwife, HCM3).

Nurses frequently experience shortages of basic supplies while delivering care, which is due to negligence in procuring and supplying stocks. They run out of the basic supplies such as catgut, oxytocin and even cord clamps:

She was bleeding, had a tear in the perineum. I couldn’t even repair that tear. There was no suture in the institution. I had to refer that woman to the hospital. (Auxiliary Nurse Midwife, HSP3).

## Discussion

This study has shown that it is vital to address issues in relation to local health governance in the provision and utilisation of health care services, and the perceptions of the community about pregnancy care, childbirth and neonatal care.

### Medically oriented primary health care

#### Healthcare confined to ‘pills’

The first theme in this paper discusses the challenges to improving perinatal survival that are caused by the medical orientation of Nepal’s PHC system. Health care in the study district has been perceived by both health providers and service users as purely the provision of medicines, mainly pills (*Tata),* and to regard whosoever provides the pills as a doctor, and health facility premises as the only platform to deliver health care. The concept of PHC, as enunciated in Alma Ata [71], advocates a social model of healthcare. The PHC approach emphasises people and communities as resources for their health rather than giving a predominant focus on ill health and hospital care. Equity and justice, community participation and ownership, coordination and collaboration with multiple sectors are the key principles underpinning a comprehensive PHC model. Primary health care workers are expected to possess the knowledge of local socio-cultural contexts and work with the people in their communities and are supposed to be the bridge between local communities and health facilities, with key responsibilities to mobilise and empower local communities towards behaviour change and health promotion [17]. However, the present study reveals that these underpinning values and scope of PHC and a PHC worker in these remote districts are undermined. Health facilities have turned into drug (medical) stores to dispense pills, and many PHC workers have reframed their position into ‘doctors’ even leaving the guard to manage births. In their ignorance and innocence, the villagers then also regard any health care service providers as ‘doctors’. Such a false image seems to be primarily reinforced through national policies and local health systems supporting a medicalised distortion of PHC, with little regards to socio-cultural views and quality childbirth being simply a slogan in policy.

#### Healthcare within the confines of a health facility

As well as the PHC workers’ false image as doctors, health care is confined within health facility premises, reflecting the policy emphasis predominantly on health facility births and use of financial incentives for women to attend [64]. Consequently, health workers simply wait for women to come to health facilities, rather than workers travelling to attend women where they need. Even the role of the health care volunteers as community mobilisers has been diminished. They have been utilised simply to push women from community care towards health facilities.

In contrast to this narrow health facility focus in the region, international and national policy state that all delivery settings (family, community/outreach and health facility) are crucial to effectively contribute to improving perinatal survival [8, 9]. Evidence suggests that family- and community-based approaches reduce both stillbirths and neonatal deaths in low- and middle-income countries [72–77]. These include interventions such as participatory women’s group mobilisation in Bolivia [77], women’s group and homebased neonatal care in India [74, 75], women’s group mobilisation in Nepal [72], community-based newborn care package in Bangladesh [76], and local stakeholders mobilisation through MNH groups in Vietnam [73]. The essence of these approaches is community mobilisation, engagement and empowerment for behaviour change. By contract, the service approach in Nepal’s remote areas may well leave women and families uninformed and desensitised about ways to improve maternal and neonatal health. There is no dialogue between health providers and the women about such issues, and even when women visit a health facility, the health providers consider that education and counselling are far less important than prescribing pills and occasionally distributing leaflets to women who are barely literate in communities where there is no culture of reading.

### Poor quality of care: lacking a socio-cultural view of childbirth and newborn care

#### Women feeling mistreated in health facilities

The second theme presented in this paper is: ‘quality of healthcare: poor acceptance, feeling unsafe and uncomfortable in health facilities’. The study explores women’s perception and experience regarding childbirth and newborn care. Previous studies from Nepal have often discussed physical distance and the absence of transportation, including lack of transport funds as key barriers in seeking and utilising care during pregnancy and childbirth [34–36, 78]. However, this study argues that women’s perceptions of feeling unsafe, uncomfortable and disrespected during childbirth are the key reasons for their low preference for, and continued poor uptake of, even basic services from local health facilities. As discussed above (theme one), the health care approach has been of a rather reductionist type, being confined to the notion of health facility buildings and pills. National policies acknowledge a socio-cultural approach [59, 63], yet, in practice, health care for women and children in these districts is dominated by a prescriptive medical model. Nepal’s long-term plan related to MNH describes access to health not just in terms of physical and financial access [60], but also socio-cultural access—women’s expectations, their dignity, trust and provider behaviour. The plan emphasises that a woman is to be understood not just as an individual, but in her socio-cultural context. Policies state that MNH programmes are to be implemented on a human rights-based approach, respecting a woman’s dignity, right to healthcare and privacy [59, 63]. This study confirms that, in these villages at least, such policy values are merely acknowledged on paper and, in practice, women, who attended health facilities, felt controlled and mistreated by the service providers. This finding is supported by a recent study from Dhaka [79], Bangladesh, which also found that women, particularly of low socio-economic status, were reluctant to attend modern health facilities for childbirth services due to social and authoritative distance with health service providers, and apprehension about medicalised childbirth in health facilities.

#### Poor acceptance of the newborn care

Nepal is among a small number of developing countries which introduced a national strategy on neonatal health relatively early (for Nepal, in 2004) [59]. However, more than a decade later, the recommended basic newborn care about bathing, breastfeeding and clothing are still not accepted in the mountain villages. Immediate and exclusive breastfeeding within an hour, skin to skin contact between mother and baby, postponing bathing the baby for at least 24 h after birth, and clothing the baby are recommended to keep babies warm and prevent hypothermia and infection [80], with such practices even more crucial for premature and low birth weight babies who are more vulnerable. Infection is one of the persistently leading causes of neonatal deaths in developing countries [5, 23], more so in underdeveloped health systems with high mortality settings, as in the present study. At a national level in Nepal, recent evidence shows that about 50% of newborn babies die due to infection [42], a figure which is likely to be even higher in the remote areas. Simple interventions such as optimal and early initiation of breastfeeding are estimated to reduce between 55 and 87% of neonatal deaths, while prevention and management of hypothermia is estimated to prevent between 18 to 42% of neonatal deaths [9]. However, in the study villages, these recommended practices do not align with what women and families consider as being culturally appropriate: breastfeeding before delivery and burial of the placenta, and the mother’s and baby’s bathing, was considered inappropriate, while bathing the baby immediately after birth with soap and cold water may well contribute to hypothermia but is considered locally appropriate to prevent skin infection, remove skin odour, make a baby’s skin look good, to wake the baby or stimulate breathing (even in an apparently stillborn baby); clothing a newborn baby was also seen as completely unnecessary. These findings strongly suggest that policies to reduce perinatal mortality must focus on more than just the availability of interventions and must ensure that health service providers work actively to become informed by the local socio-cultural contexts to fully carry out the recommendations of health policies in the way they are intended. This study also identified that newborn cultural practices are strongly rooted in villagers’ everyday life, so that health volunteers themselves, as members of the community, also follow these traditional practices rather than following or promoting the evidence-based policy recommendations which they are qualified to implement. Two points emerge from these findings: first, the importance of taking cultural determinants into consideration in formulation and implementation of any policy related to newborn and maternal health, and secondly, addressing well the quality of the health workforce and training to include community cultural issues. The importance of paying attention to a variety of cultural factors contributing to perinatal sickness and death is further discussed in two other papers: religio-cultural factors such as Karma, fate and destiny [57], and the gendered cultural context of motherhood [81].

### Health governance failures in delivery of care

The third theme in this paper centres on local health governance failures in providing effective pregnancy and childbirth services. The literature shows that there is a shift from generating epidemiological evidence on the ‘what, where and when’ of neonatal deaths and stillbirths [23, 82] towards understanding health system constraints in delivering appropriate preventive care [83]. The *BMC Pregnancy and Childbirth* series identified bottlenecks in delivering essential newborn care interventions, with country-level workshops with health professionals identifying health service delivery, human resource management and financing the most common barriers preventing service access and contributing to poor survival outcomes in developing countries [30]. The present study adds other key factors that compromise access to even basic services as being local health governance failure and lack of accountability at the local level.

#### Poorly accountable local health system

Nepal’s national policies state that every women and newborn must have an access to 24-h emergency obstetric and neonatal care (including blood transfusion, CS, neonatal resuscitation) [59, 60, 65]. Policies describe SBAs as the key providers [63], and every woman receiving free maternity care and childbirth incentives to birth in a health facility [64]. However, the women in this study who attended health facilities did not receive this support due to local health governance failures which result in a poorly supported workforce, poor implementation of the SBA strategy, health provider absenteeism, and the negligence, misuse of resources and corruption in the local health system. Previous studies have primarily indicated the need for integrated PHC in general to improve MNH in developing countries, emphasising the linking of health facilities with community-based interventions, and inter-sectoral coordination such as with education, income generation, women’s awareness and empowerment [84, 85]. However, local health system accountability is barely discussed as a factor crucial to advancing maternal and perinatal survival. As in the present study, besides an integrated approach, the poorly accountable local health system provides a breeding ground for misuse of resources and negligence and has therefore a substantial negative effect on the provision of MNH services at a local level.

#### Lack of an enabling work environment for nurses

The lack of support for nurses at the local level, who form the bulk of SBAs in rural areas, emerged as a key factor in the lack of or discontinuation of childbirth services at health facilities. Lack of support causes low retention and/or frequent absence of nurses from the workplace. Nepal’s SBA policy allocates a key responsibility to nurses and auxiliary nurses as skilled providers of pregnancy, childbirth and newborn care. However, their voices are little heard within the health system. This study shows that they are poorly supported, lacking basic equipment and medicines, receiving low level of support from male co-workers and the health facility management (also mostly males), and, on a personal level, feeling unsafe and isolated and are not provided with liveable on-site accommodation, electricity or reliable modes of communication. Therefore, as well needing local health system support to create an enabling environment for nurses, the need for co-workers’ collaboration and security to the nurses working in remote villages are important issues which require urgent attention in policy and management.

### Implications of the study

#### Revitalising the comprehensive primary health care approach

The findings discussed in this paper have strong policy and programmatic implications. They strongly suggest that it is not enough to pursue a long list of proven interventions in attempts to accelerate perinatal survival in areas which still record high mortality rates. Studies describe various interventions along the continuum of care from home to health facility and from pregnancy to the postnatal stage [8, 9]. Nepal’s national policies also describe a range of interventions to improve maternal and newborn survival, yet the implicit and explicit focus of these policies is oriented mainly towards pushing women to visit health facilities [59, 60], despite these not necessarily being functional or appropriate in practice. The socio-cultural contexts of the communities to be served have been undermined, both in formulating and translating the policy values and strategies into care for the villages. The PHC system has not been built upon, nor found ways to work with, the age-old faith/traditional healing system [51], and has overlooked the potential of success through working alongside family- and community-based approaches. As discussed above, high levels of perinatal mortality, a large proportion of and preference for homebirths, women’s and babies’ ritual confinement after birth and the continued high prevalence of faith healing in the villages [51, 57] strongly suggest the need for revitalisation of community health. It is speculated that missing the PHC approach in its totality could be one of the key factors for the low impact of Nepal’s Newborn Care Package which was piloted in the initial 10 districts [15]. Lack of a real PHC approach could also explain the relatively low progress in other areas of Nepal despite a significant impact more than a decade ago in reducing neonatal mortality by the Makawanpur trial in Nepal in 2004 (30% reduction within 30 months) [72].

#### Advocating an expanded view of health care: beyond ‘pills’, beyond ‘health facility/hospital premises’

Policy makers and programme implementers are urged to reorient the PHC system in Nepal towards an expanded system by incorporating understanding of local socio-cultural factors. Indeed, Kleinman [86] describes the health system as an extended cultural system that constitutes professional (formal), folk (traditional healing system) and popular arena (family/domiciliary), and recommends viewing the health system as a conceptual model to understand how health and sickness are produced and responded to in a society. He adds that sickness is produced within a family context, and the first response to it comes from within the family, with approximately 70 to 90% of sickness episodes usually managed within a family setting through self-care and traditional therapies. In the present study, the poorly governed health system, plus the predominance of fatalistic worldviews about perinatal deaths [51, 57], mean that villagers have no option but to rely on domiciliary/folk measures to respond to mother and baby’s sickness. In addition, limiting all health care to within the health facility premises is impracticable in the present study villages as over two-thirds of the women still give birth at home.

The WHO Commission on the Social Determinants of Health (CSDH) suggested redefining the role of health systems as health promoting systems, with the health system itself being a social determinant of health (SDH), and the health sector needing to take a stewardship role for action on SDH [87]. Rather than making people the passive recipients of medical treatment after sickness, central to the SDH approach are the engagement and empowerment of people to identify their own needs, and action towards health promotion. Internationally, the success of mobilising parents and parent groups has been proven by the ‘Born Too Soon’ movement that introduced pre-term birth as a key international policy agenda for reduction of neonatal mortality rates [88]. Mobilising and empowering parents and families is also one of the five key strategic activities outlined in the Every Newborn Action Plan [11]. Yet, in the study villages, health education and counselling-related jobs are considered by service providers as being of low value and insignificant, and supplying medicines is the only valued work, resulting them preferring to medicalise their work to gain higher prestige and leaving unqualified others to care for women. Such medicalisation/task shifting may well have devalued the core essence of community-based health care programmes from engaging and empowering communities, thus severely limiting the treatment of sick newborns and distracting considerably from the need for serious and dedicated engagement with women, families and communities to raise awareness of culturally appropriate health-promoting behaviours. The essence of the comprehensive PHC approach as put forward by the CSDH has been missed, and improvement in maternal and newborn health, which requires a whole system to function, is compromised.

#### Making local health system accountable to service delivery

The health governance failures explored in this study shows a complex interrelated situation. Inefficiency in delivery and poor quality MNH services due to poor accountability have also been found in a recent study from Bangladesh [89]. The Bangladesh study identified low levels of responsibility-taking on the part of health service providers, lack of monitoring/supervision, reduced community participation in the rural health centres, and poor coordination of government health centres within their own departments and with NGOs. In the present study, the issues surrounding workforce support and poorly functioning health facilities strongly indicate accountability failures in the local health systems which have significant negative impacts on maternal and infant survival. The research identifies a complicit nexus among health facilities, DHO and political leaders, where even local political leaders keep quiet despite knowing about such wrongful activities. The policy values describing the human rights-based approach, stating that survival is the right of every mother and every newborn [59, 60], appear only to be observed on paper so that receiving even basic health care is at the mercy of service providers. The rural, disadvantaged and barely literate women and families in the mountains are not by themselves able to question these health system failures. Therefore, this study recommends that approaches to increase the accountability of local health systems should be urgently made. Internationally, accountability in MNH has been one of the key priorities, especially after the UN Commission on Information and Accountability for Women’s and Children’s Health [90]. The focus is on the measurement of deaths and tracking of intervention coverage. Based on the findings of this study, it is argued that accountability should not just be limited to tracking outcomes (deaths and programme coverage) at a national level, but it is strongly imperative to find ways for the local health systems to more responsibly deliver the full level of quality care for every woman and every newborn including those in disadvantaged, remote and rural areas, rather than focussing simply on pushing women towards health facilities and counting health facility contact. The local health system should also be accountable to address the concerns of SBAs working in remote villages to provide a more enabling work environment—in the health facilities and villages. It is imperative that the District Health Office, local health facility boards (management committees) and male health workers collaborate to make these improvements and personally monitor them at all levels.

### Strengths and limitations

This study demonstrates that qualitative interviews are a feasible method to collect the views of women and their family members who have lost their babies. The number of participants studied provided a rich description within the themes. The post-interview chats proved useful to uncover further insight about the health service contexts shaping perinatal survival in these remote areas. The interviews were transcribed after leaving the field, although the key local terms and meanings were verified by the first author after repeated listening of audio files during the fieldwork period. Member check of transcripts later was impossible due to the remoteness of the study location (and in addition, the fieldwork being interrupted by the 2015 Nepal earthquake). To ensure translation rigour, six randomly chosen interview transcripts were checked by five researchers with expertise in both English and Nepali languages, comprising the first author’s fellow PhD students and research fellows. The main limitations of this study are that findings are context specific to Nepal’s mountain villages and there is a potential of conceptual generalisability of the key findings in other areas of Nepal, South Asian countries and countries in Africa which are still struggling with high perinatal mortality rates. Although the same health service delivery failures in the villages are likely to also be a key contributing factor to maternal deaths, it was beyond the study’s scope to also inquire into these.

## Conclusions

Addressing poor perinatal survival and continuing high perinatal mortality rates in remote mountain villages in Nepal needs coordinated consideration of local health care delivery contexts in policy development, planning and programme implementation. The local health systems of the remote regions have not been sufficiently prepared to ensure access to basic pregnancy, childbirth and postnatal care for mothers and babies, despite these being a focus of national-level policy. Healthcare quality has been diminished, with the essence of primary healthcare being severely narrowed down to the dispensing of pills, women’s experiences being unsafe and uncomfortable at health facilities, culturally misaligned newborn care practices, and failures in workforce management resulting in absenteeism and negligence, and corruption in reporting, incentives and supply management. The lives of women and children have therefore been compromised due to poorly accountable local health systems and poor consideration of socio-cultural factors in health service delivery. It is imperative to enforce basic standards in health facilities, and to sensitise women and families about the right to receive quality health care, and make nurses and health service providers accountable to service delivery with respect to women and families in their cultural context. In this regard, there is scope to conduct further in-depth studies into the nature of client-provider relationships and communication in these regions. In-depth studies about how behaviour change and health educational approaches can be successfully implemented would be equally valuable to understand the knowledge transfer process from health service providers and volunteers to disadvantaged women and families in the communities. Otherwise, the lives of mothers and babies in such areas will remain trapped in a complexity of health system challenges, all of which stand in the way of turning the national policy into reality in the remote regions in order to reduce the current significant burden of preventable neonatal deaths.

## Additional files

Additional file 1: In-depth interview guide. (DOCX 19 kb) 

Additional file 2: Collective features of study participants. (DOCX 27 kb)

## Abbreviations

CS: Caesarean Section
CSDH: Commission on Social Determinants of Health
DHO: District Health Office
DoHS: Department of Health Services
FCHV: Female Community Health Volunteer
HCM: Health Care Manager
HSP: Health Service Provider
MDG: Millennium Development Goal
MNH: Maternal and Newborn Health
MOHP: Ministry of Health and Population
NDHS: Nepal Demographic and Health Survey
ORC: Out Reach Clinic
PHC: Primary Health Care
SBA: Skilled Birth Attendant
SDG: Sustainable Development Goal
SDH: Social Determinants of Health
